# *P. hybridus* Extract Ze 339 Inhibits RSV Infection by Altering Host Metabolism

**DOI:** 10.3390/v18070697

**Published:** 2026-06-24

**Authors:** Fabian Otte, Verena M. Merk, Georg Boonen, Thomas Klimkait, Veronika Butterweck, David Hauser

**Affiliations:** 1 Department of Biomedicine, University of Basel, 4051 Basel, Switzerland; fabian.otte@unibas.ch (F.O.); thomas.klimkait@unibas.ch (T.K.); 2 Switzerland Medical Department, Max Zeller Söhne AG, 8590 Romanshorn, Switzerland; verena.merk@zellerag.ch (V.M.M.); georg.boonen@zellerag.ch (G.B.)

**Keywords:** RSV, Ze 339, *Petasites hybridus*, Butterbur, respiratory syncytial virus, antiviral, mode of action

## Abstract

Respiratory syncytial virus (RSV) remains a major cause of severe respiratory disease worldwide, particularly affecting young children and immunocompromised individuals, highlighting the need for additional therapeutic strategies. In this study, the antiviral activity of the *Petasites hybridus* extract Ze 339 was investigated in RSV-infected cell-culture models. Antiviral efficacy was assessed using plaque reduction assays, reporter virus analyses, and proteomic profiling to elucidate potential mechanisms of action. Ze 339 potently reduced the infectivity of both RSVA and RSVB in vitro and retained antiviral activity when administered up to six hours post-infection, resulting in markedly reduced plaque formation and viral protein biosynthesis without inducing cytotoxicity. Proteomic analyses revealed that Ze 339 modulates host cell pathways associated with reduced cell proliferation, attenuated immune signaling, and enhanced cholesterol and lipid metabolism. These changes were more pronounced in infected than in uninfected cells and coincided with a marked downregulation of viral proteins. The observed proteomic signature suggests a host-directed antiviral effect and identifies altered lipid metabolism and cell-cycle-associated pathways as potential contributors to reduced RSV replication. Taken together, these findings demonstrate the antiviral activity of Ze 339 against RSV and support the hypothesis that modulation of host cell pathways contributes to its antiviral effects, providing a rationale for further evaluation of Ze 339 as a repurposed therapeutic candidate for RSV infection.

## 1. Introduction

According to the World Health Organization (WHO), infection with respiratory syncytial virus (RSV) accounts for approximately 3.6 million hospitalizations per year. RSV is a negative-sense, single-stranded RNA virus (*Orthopneumovirus* genus) that causes acute lower respiratory infections [1]. Especially infants, the elderly, and immunodeficient people are at high risk for severe courses of infection. Transmission of RSV typically occurs via droplets or smear infections, leading to symptoms in the upper respiratory tract such as rhinorrhea and pharyngalgia. The propagation of the infection to the lower respiratory tract is characterized by a range of symptoms, including coughing, shortness of breath, rapid breathing, bronchospasm, and wheezing [2].

The infectious cycle of RSV can be roughly categorized into the following steps: viral attachment and entry, transcription and replication, and finally, assembly and release of new virions [1]. Infected cells can fuse with neighboring cells and form multinucleated syncytia as a pathogenic strategy that enhances, e.g., viral spread [1]. In vivo, RSV primarily infects ciliated airway epithelial cells and type I alveolar epithelial cells via the interaction of viral glycoprotein (G) and fusion protein (F) with host cell factors, such as glycosaminoglycans (GAGs) [1]. Upon membrane fusion, the viral ribonucleoprotein complex is released into the cytoplasm, where transcription and replication occur. This process is most likely to occur directly within the plasma membrane or endosomes via macropinocytosis. Viral inclusion bodies form, allowing the build-up of viral components as a hub for virion production [3]. Subsequently, new virions are assembled, and budding occurs, involving the interaction of fusion proteins with lipid rafts in the plasma membrane [4,5,6]. The RSV replication cycle follows a defined temporal pattern, with progeny virus release initiating at approximately 10–12 h post-infection. Viral mRNA levels reach a plateau between 14 and 18 h post-infection, while maximal progeny virus release is observed around 24 h post-infection [7].

To date, the European Medicines Agency (EMA) has approved three vaccines for the prevention of lower respiratory tract diseases caused by RSV in adults, including one mRNA vaccine and two protein-based vaccines. Passive immunization with monoclonal antibodies, such as Nirsevimab, is indicated in “newborns in their first RSV-season and in infants up to 24 months of age who remain susceptible to severe disease in their second RSV-season” [8]. However, apart from a limited number of antiviral small molecules (e.g., ribavirin), the general treatment strategy in most cases is to alleviate symptoms.

Despite the availability of preventive strategies, RSV continues to pose a major global health burden, causing approximately 100,000 deaths annually in children under five years of age [9]. Effective therapeutic options are therefore still urgently needed. In this context, drug repurposing represents a rational and necessary strategy to expand the spectrum of therapy options against RSV. Repurposing clinically approved compounds for new therapeutic indications could substantially accelerate regulatory approval processes, as extensive clinical and pharmacovigilance data are already available [10]. This not only reduces development time and economic costs but also has important ethical implications. Existing knowledge on safety and tolerability lowers the risk for patients and, consequently, decreases the likelihood of project failure compared with conventional de novo drug development [10].

In recent years, preclinical studies have shown that Ze 339, a subcritical CO_2_ extract of *Petasites hybridus*, a medicinal plant, exhibits antiviral activity against different SARS-CoV-2 strains [11,12]. Leaf extracts of *P. hybridus* are authorized in Switzerland for the treatment of allergic rhinitis symptoms such as itching of the nose and eyes, sneezing, rhinorrhea, and nasal congestion [13]. Interest in its antiviral activity originated from the observation that Ze 339 blocks poly I:C-induced interleukin 8 (IL-8) synthesis in human nasal epithelial cells and reduces neutrophil migration [14]. Later, antiviral activity has been confirmed in Vero E6 cells infected with different SARS-CoV-2 strains (Wuhan and Delta) by plaque assays [11]. Moreover, transcriptome analysis of SARS-CoV-2-infected normal human bronchial epithelial cells (NHBEs) treated with Ze 339 revealed a reduction in the expression of several interferons associated with antiviral immune responses [12]. Although the precise mechanism of action remains incompletely understood, these studies have highlighted the potential of Ze 339 for treating viral infections. Medicines containing herbal extracts are, in general, a complex composition of various ingredients, and a precise mode of action is therefore difficult to identify. However, for Ze 339, most of its pharmacological actions have been attributed to the activity of the sesquiterpenes petasin, isopetasin, and neopetasin, which have previously been reviewed [13]. In short, Ze 339 has been shown to inhibit the synthesis of proinflammatory mediators, such as leukotrienes, cytokines, and chemokines, which are suspected to dampen acute allergic responses and associated inflammation [13].

Herbal medicinal products are considered to have only mild side effects, and their composition of several active ingredients allows a multitarget mechanism leading to broad-spectrum efficacy. A novel herbal-based therapy approach against RSV infection could display an interesting option for early intervention, with the potential to prevent disease progression and consequently reduce the incidence of severe cases. Therefore, we here investigated the in vitro antiviral properties of the *P. hybridus* extract Ze 339 against RSV infection. By doing time-lapse inhibition experiments and by using proteomics analyses, we narrowed down the potential mechanism of action to a select number of cellular processes that influence the life cycle of RSV.

## 2. Materials and Methods

### 2.1. Compounds and Extract

Ze 339 was manufactured by Max Zeller Söhne AG, Romanshorn, Switzerland, using a patented procedure [15,16]. One coated film tablet contains 17–40 mg of Ze 339 and is standardized to 8 mg petasins. The herein used Ze 339 batch #150056 (described in [11]) contained 37.7% total petasins and 27.2% fatty acids. The remaining 35.1% contained other constituents, such as essential oils, sterols, minerals, and vitamins. Pyrrolizidine alkaloids were quantitatively removed in the manufacturing process using online adsorption technology and were no longer detectable (limit of quantification < 2 ppb). The extract was dissolved in a mixture of dimethyl sulfoxide (DMSO) and H_2_O (50:50), and a stock solution of 2 mg/mL was prepared and further diluted with assay buffer (Dulbecco’s Modified Eagle Medium [DMEM], 2% fetal bovine serum [FBS], 1% penicillin/streptomycin [Pen/Strep]) to the final concentrations of 0.039–40 µg/mL in a compound 96-well master plate for further distribution (adapted from [11]).

Ribavirin (ICN-1229) was purchased from Selleckchem. A 1 mg/mL stock solution was prepared and further diluted with assay buffer to the desired concentrations (0.006–12.5 µg/mL). To avoid cytotoxicity, the final DMSO concentration in all cellular experiments did not exceed 0.5%.

### 2.2. Cell Lines

Adenocarcinomic human alveolar basal epithelial cells expressing ACE2 and TMPRSS2 (A549-A/T) were obtained from NIBSC (A549-ACE2 Clone 8-TMPRSS2; product number 101006; Glasgow, Scotland). Cells were maintained in DMEM, high-glucose media, and were supplemented with 1% Pen/Strep (Bioconcept, Allschwil, Switzerland) and 10% FBS (Gibco, Thermofisher, Waltham, MA, USA) at 37 °C in a humidified atmosphere with 5% CO_2_ for general propagation.

### 2.3. Viruses

Clinical isolate derivatives of RSVA (ON1, 0594 strain) and RSVB (BA9, 9671 strain) were provided by M. Ludlow, University of Veterinary Medicine, Hannover, Germany [17]. Recombinant RSVB expressing eGFP was used for tracking and quantification of viral protein production. Viral titers were determined by plaque assays in A549-A/T.

### 2.4. Plaque Assay and Cytotoxicity Assessment

Antiviral activity was determined by the degree of inhibition of viral cytopathic effect (CPE) or by expression levels of eGFP. Briefly, A549-A/T cells were seeded at a density of 4 × 10^6^ cells/96-well plate one day before infection. On the day of infection, cells (80% confluent) were incubated with the above-mentioned compound concentration range. Compound was added 2 h before, at the same time, or 2–6 h after virus infection. Cells were infected with a multiplicity of infection (MOI) of 0.01 (tissue culture infectious dose [TCID_50_]) of RSVA or RSVB, and all conditions were performed in three technical replicates. As a virus inhibition control, one replicate of ribavirin, as outlined above, was included. Cells were incubated at 37 °C for either 3 days for eGFP- or 8 days for CPE-readout. For cytotoxicity, an additional plate was treated with the compound only to evaluate the compound’s effect on cellular viability for the same incubation period. To inactivate the virus, 20 μL of formaldehyde (3.7% *w*/*v*) was added for 20 min to the cultures. Fixative and culture media were aspirated, and crystal violet (0.1% *w*/*v*) (Sigma-Aldrich, Buchs, Switzerland) was added to each well and incubated for 5 min. Subsequently, the fixed and stained plates were gently rinsed several times under tap water and dried before plate reading. As the number of plaques observed per well was not easily distinguishable by eye, plates were acquired on a CTL ImmunoSpot S6 Ultimate-V Analyzer (CTL, Cleveland, OH, USA) with either brightfield or GFP acquisition mode, and the images were counted using ImageJ 1.54g software. For image processing, images were made binary according to the ImageJ definition; the limit to threshold option was enabled under ‘set measurements’, and pixels of the selected area were counted. All images were processed using the same analysis macro and identical threshold settings. The results were normalized to positive (virus-infected) controls in each assay plate.

### 2.5. Proteomic Analysis

A549-A/T cells were seeded at a density of 1 × 10^5^ cells per well 1 day before infection in a 24-well plate format. On the day of infection, 10 µg/mL of Ze 339 was added to the cells, and the cells were infected with an MOI of 0.1 (TCID_50_) with RSVB-eGFP. Twenty-four hours after infection, cells were washed twice in cold PBS, scraped off the plate, and transferred to a 1.5 mL tube. Cells were quickly collected at 500× *g* for 5 min and snap-frozen.

For LC-MS sample preparation, cells were lysed in 60 µL buffer containing 100 mM triethylammonium bicarbonate (TEAB) pH = 8.5/5% sodium dodecyl sulfate (SDS)/10 mM Tris(2-carboxyethyl)phosphine (TCEP) by 10 min heating at 95 °C followed by 10 min sonication (30 s on, 30 s off per cycle) on a Pixul^®^ system (Active Motif, Carlsbad, CA, USA) using the following settings: Pulse [N]: 50; PRF [kHz]: 1; burst rate [Hz]: 20. Protein extracts were alkylated using 15 mM iodoacetamide at 25 °C in the dark for 30 min. For each sample, 20 µg of protein lysate was captured, digested, and desalted using the SP3 approach [18] using a Fluent liquid-handling platform (Tecan Group Ltd., Männedorf, Switzerland). In brief, Speed Beads™ (#45152105050250 and #65152105050250, GE Healthcare, Chicago, IL, USA) were mixed 1:1, rinsed with water, and diluted to the 8 μg/µL stock solution. Samples were adjusted to the final volume of 80 µL, and 10 µL of the bead stock solution was added. Proteins were bound to the beads by the addition of 90 µL of 100% acetonitrile to the samples, which were then incubated for 8 min at room temperature with gentle agitation (200 rpm). Samples were placed on a magnetic rack and incubated for 5 min. Supernatants were removed and discarded. The beads were washed twice with 160 µL of 70% (*v*/*v*) ethanol and once with 160 µL of 100% acetonitrile. Samples were placed off the magnetic rack, and 50 µL of digestion mix (10 ng/µL of trypsin in 50 mM TEAB) was added. Digestion was allowed to proceed for 12 h at 37 °C. After digestion, samples were placed back on the magnetic rack and incubated for 5 min. Supernatants containing peptides were collected and dried under vacuum.

Peptides were resuspended in 0.1% aqueous formic acid, and 0.2 μg of peptides were subjected to LC–MS/MS analysis using an Orbitrap Exploris 480 Mass Spectrometer fitted with a Vanquish Neo (both Thermo Fisher Scientific, Waltham, MA, USA) and a custom-made column heater set to 60 °C. Peptides were resolved using an RP-HPLC column (75 μm × 30 cm) packed in-house with C18 resin (ReproSil-Saphir C18–AQ, 1.5 μm resin; Dr. Maisch GmbH, Ammerbuch, Germany) at a flow rate of 0.2 μL min^−1^. Separation of peptides was achieved using the following gradient: 2% Buffer B to 8% Buffer B in 5 min, 8% Buffer B to 25% Buffer B in 45 min, and 25% Buffer B to 35% Buffer B in 10 min. Buffer A was 0.1% formic acid in water, and buffer B was 80% acetonitrile and 0.1% formic acid in water. The mass spectrometer was operated in DIA acquisition mode with a total cycle time not exceeding 3 s. For MS1, the following parameters were set—resolution: 120,000 FWHM (at 200 *m*/*z*); scan range: 390–910 *m*/*z*; injection time: auto; and normalized AGC target: 300%. MS2 (SWATH) scans were acquired using the following parameters—isolation window: 10 *m*/*z*; HCD collision energy (normalized): 28%; normalized AGC target: 3000%; resolution: 15,000 FWHM (at 200 *m*/*z*); precursor mass range: 400–900 *m*/*z*; max. fill time: 22 ms; and data type: centroid.

The acquired raw files were searched using SpectroNaut (v19.0, directDIA workflow, default settings) against a human database downloaded from Uniprot (https://www.uniprot.org/taxonomy/9606, accessed on 22 February 2022) and RSV-virus database downloaded from Uniprot (https://www.uniprot.org/taxonomy/11250, accessed on 21 August 2025) using the following search criteria: full tryptic specificity was required (cleavage after lysine or arginine residues, unless followed by proline); 3 missed cleavages were allowed; carbamidomethylation (C) was set as a fixed modification; oxidation (M) and N-acetylation (N-term) were applied as variable modifications. The identified transitions were exported as tsv files and processed using the Msstats R package v4.0.1 [19].

### 2.6. Gene Ontology Enrichment and Network Analysis

Gene ontology (GO) analysis was performed using the DAVID classification system (https://davidbioinformatics.nih.gov/, accessed on 18 January 2026) [20]. Proteins significantly different between conditions were compared to all proteins detected in the proteomic screen using the GO GOTERM_BP_DIRECT annotation dataset, with a minimum number of hits set to four for overall analysis and to two for up- and downregulated subsets. GO terms were reduced using an rrvgo-based R script, with a medium (0.7) allowed similarity. Reduced terms with a minimum of 2 clustered terms and a maximum *p*-value threshold of 0.05 with Fisher’s exact test were plotted.

Network analysis was obtained using the String v12 database [21]. Each node represents a protein regulated in compound-treated samples compared to untreated controls, and each edge shows protein–protein interaction as determined by experiments and databases. Sequence homology is shown in purple, and medium confidence (0.400) was used for interaction scores.

## 3. Results

### 3.1. Ze 339 Exerts Antiviral Activity Against RSV

**Figure 1:**
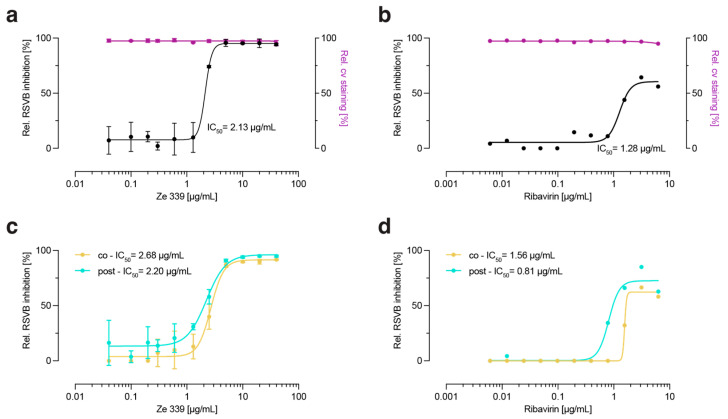
Ze 339 inhibits RSV infection independent of treatment timing. (**a**,**b**) A549-A/T cells were treated with decreasing concentrations of (**a**) Ze 339 (40–0.039 µg/mL) or (**b**) ribavirin (6.25–0.006 µg/mL) 2 h prior to RSVB infection. After 8 days, cells were fixed and stained with crystal violet (CV), and plaque areas were quantified using ImageJ. The purple line indicates CV staining of compound-treated non-infected controls. (**c**,**d**) A549-A/T cells were infected with RSVB and treated with (**c**) Ze 339 or (**d**) ribavirin either simultaneously with infection (co) or 2 h post-infection (post). Plaque formation was quantified as described above. Data in (**a**,**c**) represent mean ± SD of three technical replicates normalized to virus-only controls. (**b**,**d**) Positive controls from one technical replicate normalized to the virus-only control. IC_50_ values were determined by nonlinear regression analysis (GraphPad Prism, v10). Related CV images are shown in Supplementary Figure S1. RSVA data are shown in Supplementary Figure S2.

To investigate the antiviral activity of Ze 339 against RSV, different concentrations of Ze 339 (40 µg/mL–0.039 µg/mL) were tested for their ability to reduce cytolytic plaque formation on RSVB-infected A549-A/T cells (human lung carcinoma-derived, epithelial-like cells). Eight days post-infection, Ze 339 inhibited cytolytic plaque formation in a dose-dependent manner in RSVB-infected cells (Figure 1a, Supplementary Figure S1a). No evidence of cytotoxicity was observed at concentrations up to 40 µg/mL (Figure 1a, Supplementary Figure S1d). Comparable antiviral effects were observed with the positive control ribavirin (6.3 µg/mL–0.006 µg/mL) (Figure 1b, Supplementary Figure S1a). The calculated IC_50_ values were 2.13 µg/mL for Ze 339 and 1.28 µg/mL for ribavirin. Similar results were obtained for cells infected with RSVA with an IC_50_ value of 3.61 µg/mL (Supplementary Figures S1 and S2).

To assess whether the antiviral activity of Ze 339 depends on the timing of compound administration, time-of-addition experiments were performed, with Ze 339 added during infection (co-treatment) or 2 h post-infection. Interestingly, neither condition resulted in a reduction in the antiviral potential of Ze 339 or ribavirin (Figure 1c,d, Supplementary Figures S1b,c and S2). These results suggest that Ze 339 is unlikely to block viral entry but may affect viral biogenesis within the cell.

### 3.2. Ze 339 Alters Viral Protein Biosynthesis

**Figure 2:**
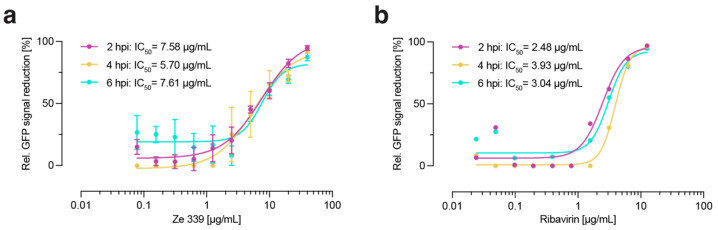
Ze 339 reduces RSVB protein biosynthesis. (**a**,**b**) A549-A/T were infected with recombinant RSVB expressing eGFP and treated with (**a**) Ze 339 (40–0.078 µg/mL) or (**b**) ribavirin (12.5–0.024 µg/mL) at 2, 4, or 6 h post-infection. After 3 days, eGFP fluorescence was quantified using ImageJ as a measure of viral protein expression. Data in (**a**) represent mean ± SD of three technical replicates normalized to virus-only controls. (**b**) Positive controls from one technical replicate normalized to the virus-only control. IC_50_ values were determined by nonlinear regression analysis (GraphPad Prism, v10). Representative fluorescence images are shown in Supplementary Figure S3.

The results of the time-of-addition experiments suggest that the mode of action of Ze 339 is associated with later stages of the RSV life cycle. To assess the effects of Ze 339 on viral protein biosynthesis, the experimental setup was adapted as follows. A549-A/T cells were infected with a recombinant RSVB strain expressing eGFP and treated with Ze 339 (40 µg/mL–0.078 µg/mL) or ribavirin (12.5 µg/mL–0.024 µg/mL) 2, 4, or 6 h post-infection. eGFP was used as a read-out marker for viral genome transcription and translation. Three days post-infection, eGFP fluorescence signal was quantified, and IC_50_ values were calculated for both compounds (Figure 2a,b, Supplementary Figure S3). Across all time points tested, Ze 339 reduced eGFP expression in a dose-dependent manner. The calculated IC_50_ values ranged from 5.70 µg/mL to 7.61 µg/mL for Ze 339 and from 2.48 µg/mL to 3.93 µg/mL for ribavirin. In accordance with the previous findings, the temporal aspect of compound addition up to 6 h after infection does not appear to exert a pronounced influence on the antiviral effect. In summary, the data demonstrate the antiviral activity against both major RSV subtypes.

### 3.3. Ze 339 Influences the Proteome in the Presence and Absence of RSV

**Figure 3:**
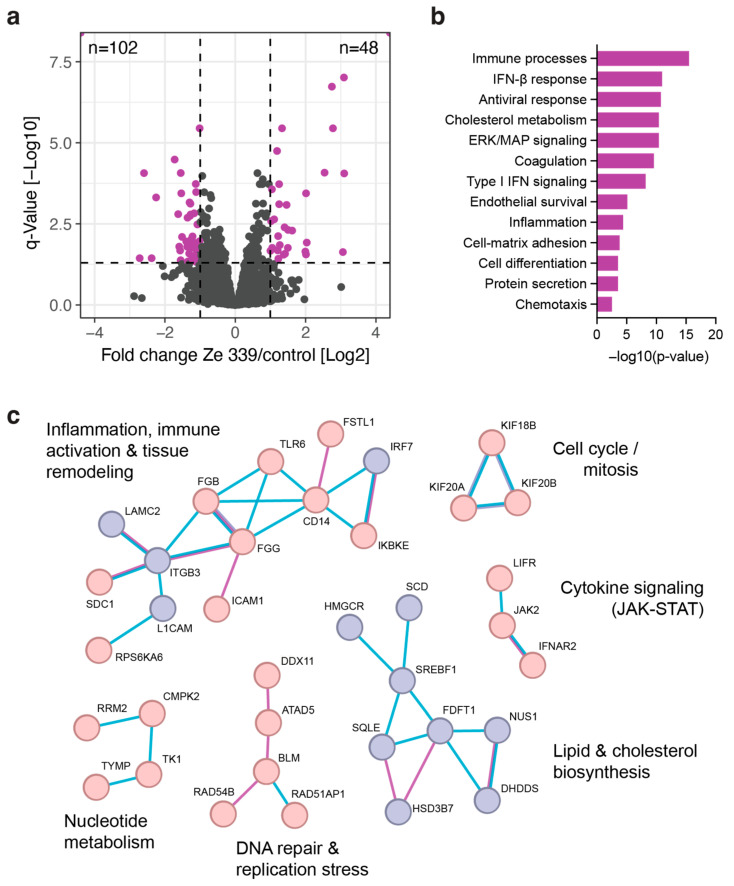
Ze 339 induces proteomic alterations in A549-A/T cells. (**a**) Volcano plot showing differentially expressed proteins in Ze 339-treated cells compared to untreated controls (*n* = 4 biological replicates). Proteins with ≥2-fold change and q-value < 0.05 are highlighted (lilac), and non-significant proteins are shown in gray. Dashed lines indicate selected thresholds. Numbers of up- and downregulated proteins are indicated (18 with positive infinite values; 59 with negative infinite values). (**b**) Gene ontology (GO) enrichment analysis of significantly regulated proteins. (**c**) STRING network analysis of regulated proteins, with upregulated proteins shown in blue and downregulated proteins in red. Edges represent associations from curated databases (blue), experimentally determined interactions (magenta), or protein homology (lilac). Directional GO enrichment analysis is shown in Supplementary Figure S4a,b).

The results from the time-of-addition experiments suggest that the mode of action of Ze 339 could be associated with the protein biosynthesis of the cells. Therefore, the proteome of cells treated with Ze 339 was analyzed by mass spectrometry. To ensure a robust antiviral response and facilitate detection of proteomic changes, a concentration of 10 µg/mL was selected, corresponding to the upper dynamic range of the antiviral dose–response curve. Cells exhibited significant changes in the expression of several proteins, with 48 proteins upregulated and 102 downregulated (Figure 3a). Gene ontology (GO) enrichment analysis of the regulated proteome revealed a significant modulation of several molecular pathways associated with immune and antiviral responses, inflammatory signaling, cell proliferation and DNA replication, and cholesterol metabolism (Figure 3b). Additional analysis by direction of regulation (Supplementary Figure S4a,b) showed that lipid and cholesterol metabolism, phospholipid efflux, and xenobiotic responses were enriched among the upregulated proteins, whereas immune, interferon, NF-κB, and cell-cycle-associated pathways were predominantly downregulated. STRING network analysis confirmed these clusters and shows a distinct association of proteins falling into the same categories as observed with the GO analysis (Figure 3c). These results suggest that the observed antiviral mechanism of Ze 339 may be associated with an unfavorable viral environment. To evaluate this hypothesis, the proteome of cells infected with RSV with or without Ze 339 treatment was also analyzed.

**Figure 4:**
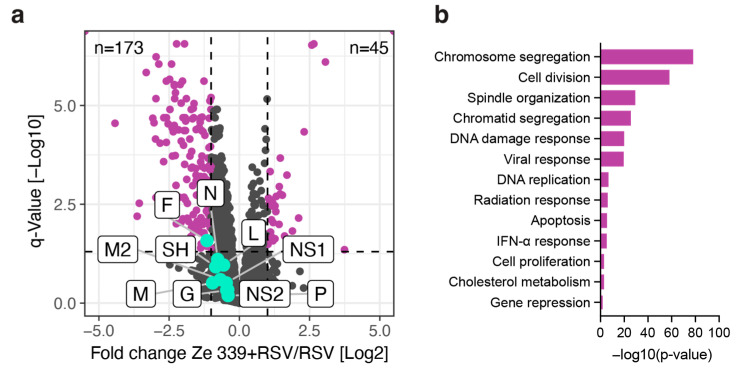
Ze 339 modulates the proteome of RSVB-infected cells. (**a**) Volcano plot showing differentially expressed proteins in Ze 339-treated, RSVB-infected cells compared to RSVB-infected controls (*n* = 4 biological replicates). Proteins with ≥2-fold change and q-value < 0.05, and RSV proteins are highlighted (lilac and cyan, respectively); non-significant proteins are shown in gray. Dashed lines indicate selected thresholds. Numbers of up- and downregulated proteins are indicated (19 with positive infinite values, 14 with negative infinite values). (**b**) GO enrichment analysis of significantly regulated proteins under infection conditions. Abbreviations: F, fusion protein; N, nucleoprotein; L, large polymerase protein; M, matrix protein; M2, matrix protein 2; SH, small hydrophobic protein; G, glycoprotein; P, phosphoprotein; NS1/NS2, non-structural proteins 1 and 2. Directional GO enrichment analysis is shown in Supplementary Figure S4c,d. Additional differential expression comparisons are shown in Supplementary Figure S5.

Cells were infected with RSVB and co-treated with 10 µg/mL of Ze 339 or infected with the virus only. While both conditions induce substantial proteome change, with more than 400 proteins significantly altered compared to cells only (Supplementary Figure S5; 2-fold change, q-value < 0.05), Ze 339-treated samples show more protein downregulation. This is also visible in direct comparison of both conditions, with all viral proteins (F, fusion protein; N, nucleoprotein; L, large polymerase protein; M, matrix protein; M2, matrix protein 2; SH, small hydrophobic protein; G, glycoprotein; P, phosphoprotein; NS1/NS2, non-structural proteins 1 and 2) tending to be downregulated in the presence of Ze 339, and the majority of differentially regulated proteins downregulated (173 vs. 45, Figure 4a). The reduced abundance of multiple RSV proteins further indicates that Ze 339 retained antiviral activity under the experimental conditions used for proteomic analysis.

Gene ontology enrichment effects of Ze 339 in the background of an RSVB infection, in line with the previous results, demonstrate an influence predominantly affecting pathways related to cell cycle and proliferation, followed by antiviral and IFN responses as well as cholesterol metabolism (Figure 4b). A more detailed analysis revealed that cholesterol and lipid metabolism, ER-stress-induced apoptosis, epidermal development, and steroid hormone signaling were enriched among upregulated proteins, whereas proteins involved in the cell cycle, immune responses, and immune signaling were downregulated (Supplementary Figure S4c,d). This suggests a mode of action that is independent of the classical, active defense strategy of cells.

## 4. Discussion

Worldwide, RSV continues to cause severe respiratory diseases, particularly in young children and immunocompromised individuals, underscoring the need for additional therapeutic options [2]. In this study, we demonstrate that the *P. hybridus* extract Ze 339 potently reduces RSV infectivity in vitro and retains antiviral activity even when administered after infection. Both RSVA and RSVB were efficiently inhibited, with a strong reduction in plaque formation and impaired viral protein biosynthesis up to six hours after infection. In line with previous observations, Ze 339 did not induce cytotoxic effects even at high concentrations [11,12] and, based on all available preclinical and clinical data, the safety profile of Ze 339 is considered to be well established and highly favorable, supporting its use as a promising candidate for further investigation beyond allergic rhinitis therapy [13].

Proteomic analyses suggest that the antiviral activity of Ze 339 is associated with the modulation of three major cellular pathways: (i) reduced cell proliferation, (ii) attenuation of immune signaling, and (iii) enhanced cholesterol and lipid metabolism. Although a direct connection between these pathways and viral components has not been elucidated, the treatment with Ze 339 in the context of viral infection resulted in a pronounced downregulation of viral proteins, consistent with the reduced eGFP expression observed upon treatment. Notably, the compound-induced cellular changes were more pronounced in infected than in uninfected cells, suggesting that Ze 339 induces a proteomic profile that differs from previously described senescence-associated signatures, especially during viral infections. Senescence is a stress-induced, irreversible cell-cycle arrest that was originally attributed to progressive telomere shortening [22]. However, it is now evident that other factors, such as oncogene activation, genotoxic stress, and probably also innate immune signaling pathways, eventually induce premature senescence [22]. Typical senescence markers are cell-cycle arrest, increased levels of proinflammatory cytokines, lysosomal activity, DNA damage, and oxidative stress [22]. Importantly, RSV infection has previously been shown to induce signs of senescence in vitro in cultured cells and in vivo in mouse epithelial lung tissue, with speculations that senescence induced by oxidative stress and subsequent DNA damage can have favorable or negative pathological consequences [22,23]. While the viability loss of individual cells within organs can be compensated for, reversible cellular adaptations that limit viral replication may be more favorable for maintaining organ function. In this context, the pathways identified in this study, including a downregulated pro-inflammatory and innate immune response, are consistent with a cytostatic-like proteomic signature characterized by reduced proliferation and altered immune signaling.

Consistent with this interpretation, Ze 339 treatment in uninfected cells was associated with the downregulation of immune signaling pathways and canonical inflammatory responses. Both GO and STRING analyses revealed reduced cytokine signaling (e.g., JAK2, IFNAR2) and interferon responses. In RSVB-infected cells, antiviral immune responses were attenuated upon Ze 339 treatment, most prominently affecting type I interferon responses, cytokine production, and general antiviral defense pathways. These findings align with previous observations in SARS-CoV-2-infected NHBEs, where transcriptomic analyses demonstrated a strong reduction in interferon (IFN)-related gene expression, including IFNA10, IFNA7, and IFNL3, following Ze 339 treatment in infected cells [12]. However, a mild increase in IFNA7 and IFNL3 expression in uninfected cells was reported for Ze 339 [12], an effect that was not observed in the present study.

At first glance, the attenuation of antiviral immune response may appear counterintuitive in the context of viral infection. However, the antiviral efficacy observed in our study, together with the reduction in viral protein expression, argues against a proviral effect of diminished immune signaling. Any effect on reduced immune signaling may therefore be less impactful than the effect of a Ze 339-induced cellular state less permissive to viral replication. Additionally, as RSV pathogenesis is not solely driven by viral replication but is also induced by excessive inflammatory responses and interferon-mediated tissue damage, downregulating these pathways might have beneficial effects [24,25].

While altered immune signaling and reduced cell proliferation likely generate an unfavorable environment for viral amplification, an additional antiviral mechanism of Ze 339 may involve modulation of cholesterol and lipid-associated pathways. RSV strongly depends on extensive interaction with host cell membranes during both entry and exit. RSV fusion has been shown to occur at cholesterol-rich membrane microdomains (lipid rafts) at the plasma membrane, while alternative entry routes include macropinocytosis followed by fusion within endosomes. Both entry modes are highly dependent on lipid composition and cholesterol homeostasis [1,26,27,28]. Consistent with this, cholesterol depletion has been reported to impair RSV infection in NHBE cells [27]. Moreover, the interference with host lipid metabolism, such as statin treatment, inhibits RSV replication through cholesterol- and isoprenoid-mediated effects [28].

In our proteomic analysis, Ze 339 treatment resulted in the upregulation of lipid and cholesterol metabolic enzymes, many of which represent rate-limiting steps in cholesterol biosynthesis and turnover (e.g., HMGCR, SQLE, NUS1, or FDFT1), as well as phospholipid efflux. These changes indicate altered cholesterol homeostasis and membrane lipid metabolism. The upregulation of HMGCR, SQLE, FDFT1, and NUS1 is consistent with the activation of compensatory cholesterol biosynthesis pathways that may arise in response to altered intracellular cholesterol availability. Such alterations could impair the assembly of viral replication complexes, disrupt envelope formation and maturation, and interfere with viral budding and release. This ultimately may lead to reduced viral infectivity and syncytium formation. In addition, the attenuation of interferon-associated signaling raises the possibility that immune and lipid metabolic pathways are mechanistically linked, potentially through interactions between STAT- and SREBP-dependent regulatory networks. Taken together, these findings support a working hypothesis in which Ze 339 creates a cellular lipid environment that is less favorable for RSV replication, assembly, and transmission. A limitation of the present study is the use of a single transformed epithelial cell line. Consequently, the proteomic changes observed may partly reflect cell line-specific responses. Furthermore, the antiviral dose–response experiments were performed using technical replicates within representative experiments. Although consistent antiviral effects were observed across independent assay formats and against both RSV subtypes, future studies in RSV-relevant model systems, including primary airway epithelial cultures and in vivo models, will be required to validate these findings.

The antiviral properties of various natural products have long been recognized; however, the evidence is limited to preclinical observations [29]. To date, there are no authorized herbal medicinal products that are indicated for the treatment of viral infections. The use of Ze 339 appears to be a promising option, as it is an approved over-the-counter (OTC) medication in Switzerland for the treatment of allergic rhinitis symptoms. Clinical studies and real-world data (RWD) confirm a favorable safety profile with only one adverse event in 469,500 defined daily doses [13]. The side effects that occurred were predominantly mild gastrointestinal complaints [13]. In general, *P. hybridus* contains toxicological critical amounts of pyrrolizidine alkaloids (PAs) and their N-oxide derivatives [30]. These substances are part of the plants’ defense strategy against herbivores but may cause liver damage in humans. In the context of herbal medicines, it is crucial to ensure a minimum PA content through strict quality controls and sensitive analysis methodologies such as ultra-high performance liquid chromatography–time-of-flight mass spectrometry (UPLC-TOF-MS) to mitigate the risk of liver damage [31]. For example, these analyses confirmed that PAs are effectively removed in the CO_2_ extraction process for the *P. hybridus* leaf extract Ze 339 [31]. In addition, Ze 339 is manufactured according to predefined quality specifications and batch-release criteria to ensure consistent composition and product quality across production batches. Besides antiallergic effects, petasins from *P. hybridus* have also been shown to exhibit spasmolytic effects [32]. Therefore, extracts from *P. hybridus* root are also used to treat gastrointestinal complaints [32]. Besides the previously outlined antiviral effects [11,12], preclinical studies have demonstrated the potential of petasins for anti-adipogenic effects [33] and selective anti-tumor activity in colon carcinoma models [34,35]. In addition to the results of this study, these findings highlight the potential for repurposing *P. hybridus*–containing drugs to broaden their range of medical applications, with RSV as a clinically very relevant disease. Although herbal medicines generally have a lower range of adverse effects, they cannot be considered a substitute for vaccinations, particularly in high-risk groups. However, the antiviral activity observed in the present study supports further investigation of the *P. hybridus* extract Ze 339 as a potential candidate for early intervention strategies against RSV infection.

## 5. Conclusions

In conclusion, the present study demonstrates robust antiviral activity of the *P. hybridus* extract Ze 339 in RSV-infected cells and delineates a potential mode of action via altered lipid metabolism and cytostatic effects. The data show that Ze 339 influences the proteome towards a less permissive state by downregulating pathways associated with cell proliferation, thereby limiting viral replication. This is consistent with observations that altered lipid metabolism prevents RSV from fusing with host membranes and releasing new virions. Future studies employing dedicated functional assays and targeted validation of key proteins will be necessary to elucidate the underlying molecular mechanisms. Taken together, this study suggests a likely mechanism of action in the antiviral activity of Ze 339. This could be the first step towards repurposing drugs containing *P. hybridus* extracts for antiviral use and gaining approval for other indications. However, further research is required to confirm these effects in complex organisms and to determine whether these findings translate beyond in vitro systems.

## Data Availability

Raw proteomics data are deposited in the ProteomeXchange Consortium under accession number PXD079925 (MassIVE accession: MSV000102215). All other data presented in this study are available on request from the corresponding authors.

## Supplementary Materials

The following supporting information can be downloaded at https://www.mdpi.com/article/10.3390/v18070697/s1; Figure S1: Plaque formation assay plates; Figure S2: Ze 339 inhibits RSVA infection; Figure S3: eGFP fluorescence images; Figure S4: Directional GO enrichment analysis; Figure S5: Additional differential expression analyses.

## Abbreviations

The following abbreviations are used in this manuscript:

A549-A/T	Adenocarcinomic human alveolar basal epithelial cells expressing ACE2 and TMPRSS2
CPE	Cytopathic effect
CV	Crystal violet
DMEM	Dulbecco’s Modified Eagle Medium
DMSO	Dimethyl sulfoxide
eGFP	Enhanced green fluorescent protein
EMA	European Medicines Agency
ERK	Extracellular signal-regulated kinase
F	Fusion protein
FBS	Fetal bovine serum
G	Glycoprotein
GAG	Glycosaminoglycan
GO	Gene ontology
IFN	Interferon
IFNAR	Interferon-alpha/beta receptor
IL-8	Interleukin 8
JAK	Janus kinase
L	Large polymerase protein
M	Matrix protein
M2	Matrix protein 2
MAP	Mitogen-activated protein
MOI	Multiplicity of infection
N	Nucleoprotein
NHBE	Normal human bronchial epithelial cells
NS1/NS2	Nonstructural proteins 1 and 2
OTC	Over-the-counter
P	Phosphoprotein
PA	Pyrrolizidine alkaloid
Pen/Strep	Penicillin/streptomycin
RBV	Ribavirin
RSV	Respiratory syncytial virus
RWD	Real-world data
SDS	Sodium dodecyl sulfate
SH	Small hydrophobic protein
SREBP	Sterol regulatory element-binding protein
STAT	Signal transducer and activator of transcription proteins
TEAB	Triethylammonium bicarbonate
TCEP	Tris(2-carboxyethyl)phosphine
TCID	Tissue culture infectious dose
UPLC-TOF-MS	Ultra-high performance liquid chromatography–time-of-flight mass spectrometry
WHO	World Health Organization
