# Supplementary material for: P. hybridus Extract Ze 339 Inhibits RSV Infection by Altering Host Metabolism
Source: Viruses. 2026 Jun 24;18(7):697. doi: 10.3390/v18070697 (PMC13431551; doi:10.3390/v18070697)
Supplement: Supplementary file 1 [file viruses-18-00697-s001.zip › viruses-4334810-supplementary.pdf]

Supplementary Information for

P. hybridus Extract Ze 339 Inhibits RSV Infection by Altering Host Metabolism

Fabian Otte, Verena M. Merk, Georg Boonen, Thomas Klimkait, Veronika Butterweck*, David Hauser*
*Corresponding authors: veronika.butterweck@zellerag.ch, david.hauser@unibas.ch

This PDF file includes:

Supplementary Figures S1 to S5:

Suppl. Fig. S1: Plaque formation assay plates (related to Fig. 1).

Suppl. Fig. S2: Ze 339 inhibits RSVA infection (related to Fig. 1).

Suppl. Fig. S3: eGFP fluorescence images (related to Fig. 2).

Suppl. Fig. S4: Directional GO enrichment analysis (related to Figs. 3 and 4).

Suppl. Fig. S5: Additional differential expression analyses (related to Fig. 4).

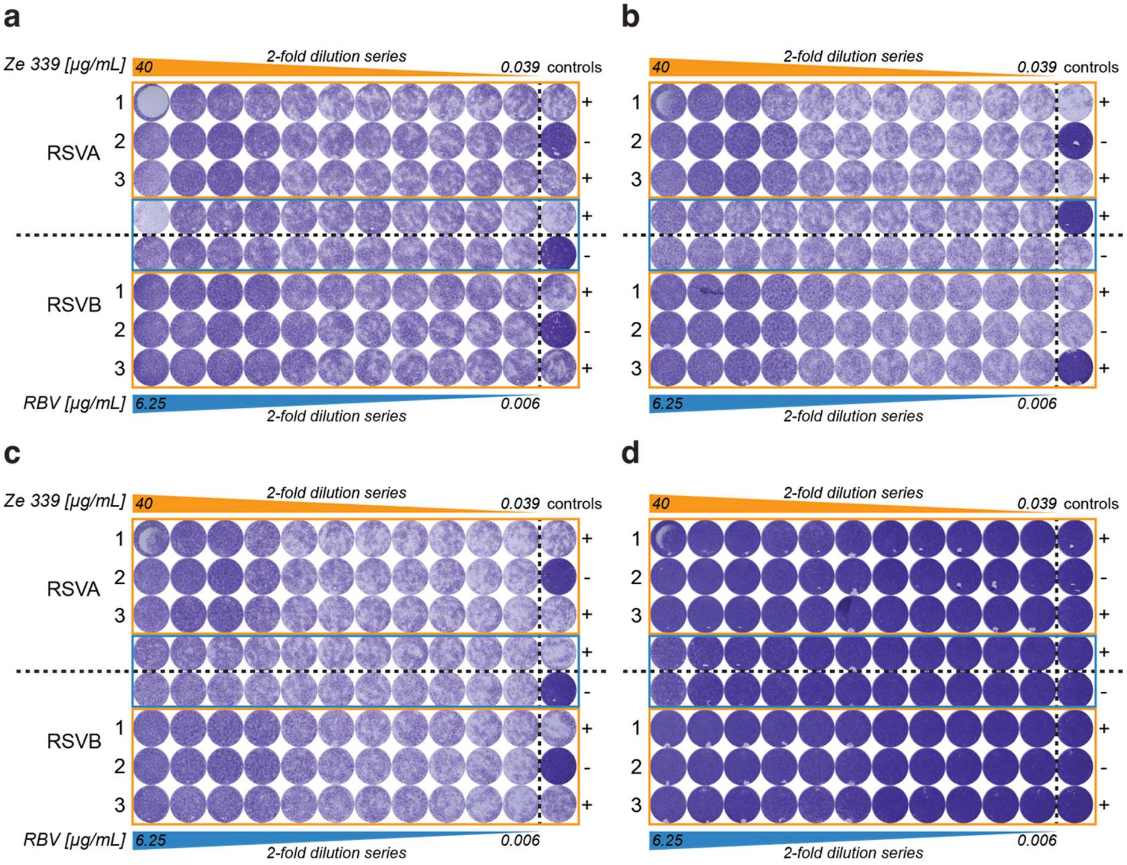

Suppl. Fig. S1: Plaque formation assay plates (related to Fig. 1).

(a-c) Crystal violet-stained plates used for plaque quantification 8 days after infection. Ze 339 or ribavirin (RBV) was added **(a)** 2 h before, **(b)** simultaneously with, or **(c)** 2 h after infection. Concentrations and dilution series are indicated for three replicates for Ze 339 (orange boxes) and for one replicate for ribavirin (blue boxes) for both RSVA and RSVB. Virus-only controls (+) and cell-only controls (-) are indicated. **(d)** Cytotoxicity control in the absence of virus.

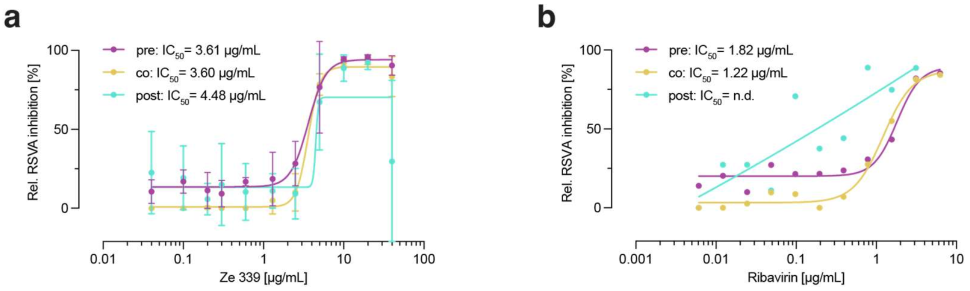

Suppl. Fig. S2: Ze 339 inhibits RSVA infection (related to Fig. 1).

(a, b) A549-A/T cells were infected with RSVA and treated with **(a)** Ze 339 (40 – 0.039 µg/mL) or **(b)** ribavirin (6.25 – 0.006 µg/mL) 2 h before infection (pre), simultaneously (co), or 2 h post-infection (post). After 8 days, plaque formation was quantified following crystal violet staining. Data represent mean ± SD of **(a)** three technical replicates or **(b)** positive control normalized to the virus-only controls. IC_{50} values were calculated by nonlinear regression analysis. Images used for quantification are shown in Supplementary Figure S1.

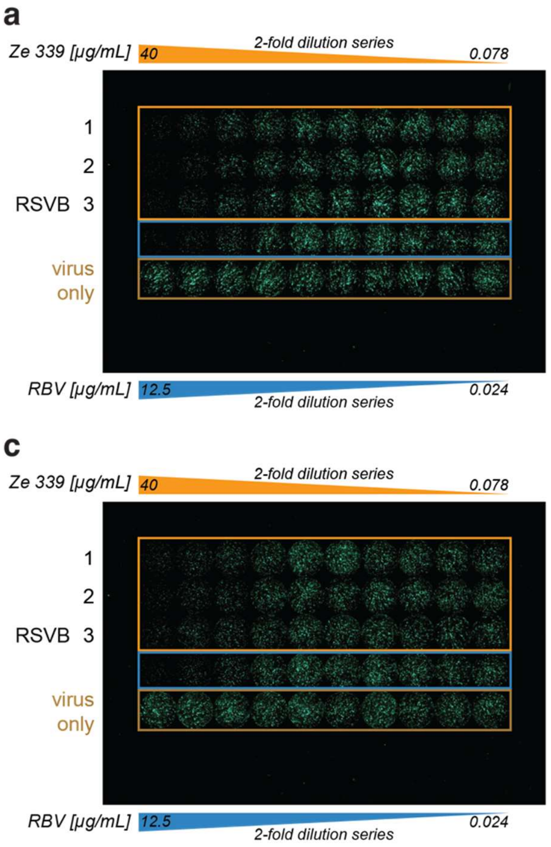

Suppl. Fig. S3: eGFP fluorescence images (related to Fig. 2).

(a-c) Fluorescence microscopy images of RSVB-infected A549-A/T cells treated with Ze 339 or ribavirin (RBV) at **(a)** 2, **(b)** 4, and **(c)** 6 h post-infection. Images were acquired 3 days post-infection. Concentrations are indicated; three technical replicates are shown for Ze 339 (orange), and one representative replicate for ribavirin (blue). Virus-only controls are shown in brown.

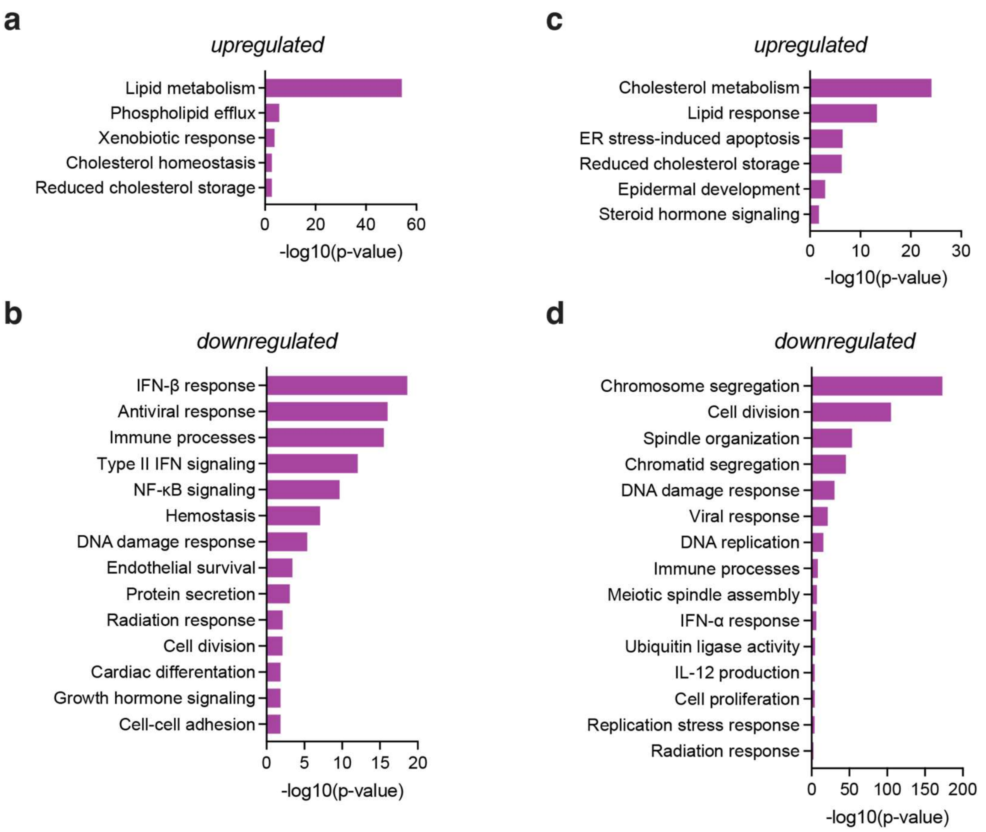

Suppl. Fig. S4: Directional GO enrichment analysis (related to Figs. 3 and 4).

(a, b) Directional GO enrichment of up- and downregulated proteins in Ze 339-treated cells compared to untreated controls. **(c, d)** Directional GO enrichment of up- and downregulated proteins in Ze 339-treated, RSVB-infected cells compared to RSVB-infected cells. Analysis was based on proteins showing ≥2-fold regulation with q-value < 0.05 (*n* = 4 biological replicates).

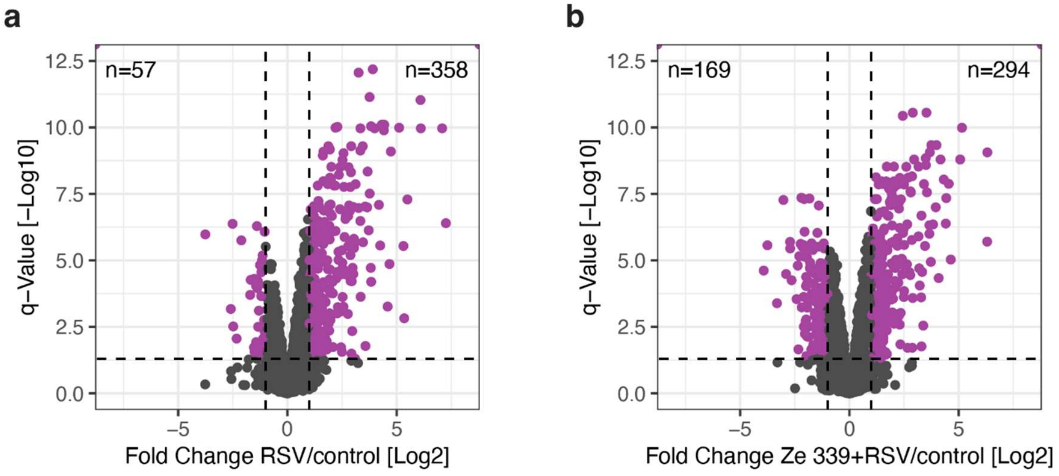

Suppl. Fig. S5: Additional differential expression analyses (related to Fig. 4).

(a, b) Volcano plots showing differential protein expression (*n* = 4 biological replicates) for **(a)** RSVB-infected cells compared to uninfected controls and **(b)** RSVB-infected, Ze 339-treated cells compared to untreated controls. Lilac dots indicate proteins with 2-fold regulation and q-value < 0.05; grey dots indicate non-significant proteins. The numbers of up- and downregulated proteins are indicated (a: 44 with positive infinite values; 14 with negative infinite values; b: 43 with positive infinite values; 24 with negative infinite values).
